# Biochemical Markers of Spontaneous Preterm Birth in Asymptomatic Women

**DOI:** 10.1155/2014/164081

**Published:** 2014-01-19

**Authors:** Ronna L. Chan

**Affiliations:** Department of Epidemiology, Gillings School of Global Public Health, The University of North Carolina at Chapel Hill, CB 7435, McGavran-Greenberg Hall, Chapel Hill, NC 27599-7435, USA

## Abstract

Preterm birth is a delivery that occurs at less than 37 completed weeks of gestation and it is associated with perinatal morbidity and mortality. Spontaneous preterm birth accounts for up to 75% of all preterm births. A number of maternal or fetal characteristics have been associated with preterm birth, but the use of individual or group biochemical markers have advanced some of the understanding on the mechanisms leading to spontaneous preterm birth. This paper provides a summary on the current literature on the use of biochemical markers in predicting spontaneous preterm birth in asymptomatic women. Evidence from the literature suggests fetal fibronectin, cervical interleukin-6, and *α*-fetoprotein as promising biochemical markers in predicting spontaneous preterm birth in asymptomatic women. The role of gene-gene and gene-environment interactions, as well as epigenetics, has the potential to further elucidate and improve understanding of the underlying mechanisms or pathways of spontaneous preterm birth. Refinement in study design and methodology is needed in future research for the development and validation of individual or group biochemical marker(s) for use independently or in conjunction with other potential risk factors such as genetic variants and environmental and behavioral factors in predicting spontaneous preterm birth across diverse populations.

## 1. Introduction

Preterm birth is defined as birth at less than 37 completed weeks of gestation and it accounts for approximately 75% of perinatal mortality and nearly half of the cases of long-term neurologic morbidity [1]. The rate of preterm births in the United States was 12.8% in 2006, a rate that had increased by more than 20% between 1990 and 2006 [2]. The estimated annual health care cost due to preterm birth had exceeded US $26 billion in 2005 or $51,600 per infant [3]. Preterm birth is a heterogeneous phenotype with many biological pathways [3] and is classified into two broad categories, spontaneous and indicated, based on the presence or absence of factors that place the mother or the fetus at risk [4–6]. Spontaneous preterm birth (sPTB) accounts for the majority of preterm births in developed countries and it occurs as a result of spontaneous onset of labor (40% to 45%) or preterm premature rupture of fetal membranes (25% to 30%) before 37 weeks of gestation. Preterm births that are the result of conditions that directly threaten the health of the mother or fetus are categorized as indicated preterm births and account for the remaining 25% to 30% of preterm deliveries [4–7].

The onset of preterm labor is thought to be brought on by multiple mechanisms or pathways that may have been initiated weeks to months before the actual presence of clinical symptoms [3, 8]. Various maternal or fetal demographic, behavioral, and clinical characteristics have been associated with preterm birth including maternal race/ethnicity [9, 10], younger maternal age [11, 12], maternal age over 35 years [13], cigarette smoking [14, 15], low prepregnancy weight [15–17], psychosocial stress [18], previous preterm birth [19], and intrauterine infection [20, 21].

The identification of risk factors for predicting preterm birth is advantageous because it allows for the initiation of risk-specific treatment for at-risk women and these risk factors may provide insights into a better understanding of the mechanisms leading to preterm birth [22, 23]. However, methods for identifying women at risk of preterm birth by the reliance of demographic, behavioral, and biological risk factors have low sensitivities [3]. Cervicovaginal fluids, amniotic fluid, urine, saliva, and serum or plasma are biologic fluids that have been used as a source for identifying biochemical markers for the prediction of preterm birth. For large-scale, population-based clinical and epidemiological studies, methodological and ethical considerations are critical as researchers choose to incorporate the sampling of biological specimens in otherwise asymptomatic women and such considerations include cost, ease of specimen collection and storage, and potential maternal and fetal risks. This paper summarizes the current literature on biochemical markers of sPTB in asymptomatic women, in which the focus is placed solely on markers that offer a great degree of acceptability to most pregnant women while minimizing maternal and fetal risks. Thereby, biochemical markers that are collected through invasive procedures such as amniocentesis are omitted from this review.

## 2. Common Biological Pathways to Preterm Birth

Biomarkers of sPTB are divided into mechanistic pathways and they include intrauterine infection and inflammation, extracellular matrix degradation, fetal stress, fetal anomalies, and estrogen metabolism pathways (Table 1).

### 2.1. Intrauterine Infection and Inflammation Pathway

#### 2.1.1. Bacterial Vaginosis

Bacterial vaginosis (BV) is an alteration of the maternal vaginal flora in which normal lactobacilli are replaced by Gram-negative anaerobic bacteria [24] and diagnosed by Gram-stain Nugent score or by the Amsel criteria. Positive results of BV are marker of intrauterine infection in asymptomatic women [20] and the presence of BV during pregnancy is consistently associated with a twofold increase in risk of spontaneous preterm birth [5, 25].

#### 2.1.2. Inflammatory Response

Intrauterine infection is an important mechanism leading to preterm birth [20]. Bacterial invasion of the choriodecidual space activates the production of a number of cytokines and these markers of inflammation (interleukins [IL] 1, 2, 6, and 8, tumor necrosis factor-*α* [TNF-*α*], and C-reactive protein [CRP]) have been evaluated as biomarkers in subsequent sPTB.

*Interleukin-6.* IL-6, a proinflammatory cytokine, is a major mediator of host response to inflammation and infection. It can be analyzed from samples of maternal cervical fluids or serum and to date, IL-6 is one of the most well-studied biomarkers of sPTB [26]. In a nested case-control study of asymptomatic women matched for race, parity, and study center [27], cervical IL-6 concentrations measured at 24 weeks of gestation were elevated in women who delivered at <32 weeks of gestation (247 ± 365 versus 84 ± 129 pg/mL) and at <35 weeks of gestation (212 ± 339 versus 111 ± 186 pg/mL) compared to women who delivered at term. Furthermore, IL-6 concentrations for women who delivered within 4 weeks of specimen collection (384 ± 444 pg/mL) were statistically significantly higher than those in their matched control (97 ± 163 pg/mL). In a meta-analysis [28], an increased risk of sPTB at <37 weeks of gestation in asymptomatic women was associated with elevated levels of cervical IL-6 (OR = 3.1, 95% CI: 2.0, 4.7). Results from a study by Paternoster et al. [29] further supported cervical IL-6 as a powerful indicator for sPTB among asymptomatic pregnant women. Paternoster and colleagues reported that concentrations of IL-6 at the 90th percentile or greater increased the risks for sPTB at <32 weeks' gestation (OR = 4.3, 95% CI: 1.2, 14.7), at <35 weeks of gestation (OR = 5.4, 95% CI: 1.8, 16.6), and at <37 weeks of gestation (OR = 3.8, 95% CI: 1.2, 12.1).

Evidence in the literature to date has not shown serum IL-6 to be a useful biomarker in predicting sPTB for asymptomatic women. Paternoster et al. [29] and Kramer et al. [30] found no association between maternal serum IL-6 around 24 weeks of gestation and sPTB, while Goldenberg et al. [31] observed only weak effects on maternal serum concentrations of IL-6 at 24 weeks of gestation on sPTB at <32 weeks' (OR = 1.3) and at <35 weeks of gestation (OR = 1.1) in a nested case-control study of asymptomatic women. A positive association was seen between elevated serum IL-6 concentrations and sPTB in a meta-analysis (OR = 1.5, 95% CI: 0.7, 3.0) [28]; however, this imprecise finding must be interpreted with caution because only two studies contributed to the subgroup analysis.

*C-Reactive Protein*. C-reactive protein (CRP) is a maternal systemic inflammatory marker that has been evaluated in the literature as a potential marker for preterm birth. In a prospective nested case-control study, Hvilsom et al. [32] examined maternal serum C-reactive protein (CRP) in early second trimester (14–18 weeks) in relation to sPTB in asymptomatic women. CRP concentrations ranged from 5.6 *μ*g/mL (at 75th percentile) to 16.4 *μ*g/mL (at 95th percentile), and elevated CRP concentrations in maternal serum (≥85th percentile) increased the risk of sPTB (OR = 2.0, 95% CI: 1.2, 3.5) compared to lower CRP concentrations (<85th percentile). In a subgroup analysis, women without a previous preterm birth but with CRP levels at the 90th percentile and the 95th percentile had an increased risk of sPTB (OR = 2.1, 95% CI: 1.1, 4.3 and OR = 2.2, 95% CI: 0.9, 5.5, resp.) compared to women with CRP levels at <75th percentile. Catov et al. [33] reported an increased risk of early to moderate sPTB (<34 weeks of gestation; OR = 2.8, 95% CI: 1.1, 7.5) and late sPTB (34 weeks to <37 weeks of gestation; OR = 2.6, 95% CI: 1.3, 5.5) for the presence of early pregnancy serum CRP (≥8 *μ*g/mL obtained <21 weeks of gestation) in asymptomatic women. Likewise, Riboni and colleagues [34] found an increase in risk of sPTB (OR = 3.1, 95% CI: 1.4, 6.8) in elevated serum CRP concentrations (≥8.4 *μ*g/mL) collected in mid-second trimester. Other studies, in contrast, showed weak [28] to no effects [30] of elevated serum CRP levels in predicting sPTB among asymptomatic women; thereby, not corroborating results from earlier research.

*Other Inflammatory Cytokines, Tumor Necrosis Factor- α*. In addition to the evaluation of IL-6 and CRP in predicting subsequent preterm birth, few earlier studies also evaluated the association between other common cytokines IL-1, IL-2, IL-8, and TNF-*α* but there was no evidence suggesting that elevated cytokine values were associated with an increased risk of sPTB [29–31, 34].

### 2.2. Extracellular Matrix Degradation Pathway

#### 2.2.1. Fetal Fibronectin

The presence of fetal fibronectin, thus far, is the most effective biochemical marker for predicting preterm birth [31, 35–39]. Fetal fibronectin, a stable glycoprotein produced by the fetal membranes, adheres the fetal membranes and placenta to the uterine lining [22, 40] and plays a critical role in facilitating the physiological separation of the placenta from the uterus after delivery [41]. Although fetal fibronectin is generally not present at levels >50 ng/mL between 16 and 22 weeks of gestation, earlier studies have shown that its presence (>50 ng/mL) in the cervix or vagina from 22 weeks of gestation and beyond is a powerful predictor of subsequent sPTB [31, 35, 36, 38, 39, 42, 43]. Goldenberg et al. [39] demonstrated that screening asymptomatic women for the presence of cervical fetal fibronectin at 24 or 26 weeks of gestation had a high sensitivity in predicting more than 60% of sPTB within the next 4 weeks (Sensitivity = 0.63, 95% CI: 0.4, 0.8; relative risk = 59.2, 95% CI: 35.9, 97.8) compared to women with a negative fetal fibronectin test (<50 ng/mL); however, this association was weaker at 28 and 30 weeks of gestation and for later gestational ages. In a related study [38], 29% of women were tested positive for cervical or vaginal fetal fibronectin if the last fetal fibronectin test was positive and 42% had a subsequent positive test result if two previous tests were positive; however, only 3% of the next fibronectin tests were positive if the previous fetal fibronectin test was negative. Data from this study not only demonstrated the presence of a positive fetal fibronectin test result predicted subsequent positive test results, but also provided additional evidence that the greater number of positive fetal fibronectin results is associated with an increased risk of sPTB at <35 and <37 weeks of gestation. Morrison and colleagues [42] also demonstrated the utility of cervical fetal fibronectin as an assessment tool in predicting sPTB among high risk asymptomatic women. Despite a small sample size, data from this prospective cohort study showed that women with positive fetal fibronectin (>50 ng/mL) had an increased risk of sPTB prior to 34 weeks of gestation (relative risk = 3.8, 95% CI: 1.5, 9.4) compared to women with negative fetal fibronectin; the risk of sPTB was further magnified when positive fetal fibronectin was coupled with positive uterine contraction (relative risk = 27.0, 95% CI: 8.7, 84.1). Similarly, a positive cervical fetal fibronectin (>50 ng/mL) at 24 weeks' gestation was found to be associated with sPTB at <32 weeks of (odds ratio [OR] = 7.6, 95% CI: 3.0, 19.2) and at <35 weeks of gestation (OR = 6.0, 95% CI: 2.4, 14.6) compared to women with negative results [29]. In a cohort study of asymptomatic pregnant women, Roman et al. [43] reported high negative predictive values and specificities for vaginal fetal fibronectin in predicting sPTB within 14 and 21 days of assessments and for sPTB at <34 weeks of gestation.

The association between the presence of fetal fibronectin and sPTB in asymptomatic women was further examined in a meta-analysis by Honest et al. [44]. The likelihood ratio, an indication of how much a given fetal fibronectin test result increases or decreases the probability of having sPTB, was 4.0 (95% CI: 2.9, 5.5) for positive result of predicting birth before 34 weeks of gestation among asymptomatic women [44].

The fetal fibronectin test can be performed as a single test or in series. A sample is collected from the posterior vaginal fornix and cervix with a sterile swab and the sample is then placed in a buffer solution and shipped to the laboratory for assay. Ideally, this test should be used in clinical settings in conjunction with patient medical history and other clinical information to assess overall risk of sPTB.

### 2.3. Fetal Stress Pathway

#### 2.3.1. Corticotropin-Releasing Hormone

Corticotropin-releasing hormone (CRH) is expressed by the human placenta and the fetal membranes, and its highest level of production is seen during the third trimester [45]. In a retrospective examination of plasma CRH levels from four gestational age intervals, Berkowitz et al. [46] found no difference in the overall mean CRH among asymptomatic women who delivered preterm and the mean CRH among women who delivered at term. Compared with term births, gestational age-specific CRH levels were not associated with predicting subsequent sPTB.

In contrast, analysis from a nested case-control study of 254 asymptomatic women showed that elevated maternal plasma CRH (>90th percentile) at 28 weeks of gestation was associated with sPTB at <35 weeks (OR = 3.5, 95% CI: 1.0, 10.9) but high levels of CRH at 24 or 28 weeks of gestation did not predict sPTB at <32 weeks of gestation or <37 weeks of gestation [47]. A study by Goldenberg et al. [31] further demonstrated similar trends in the association between CRH levels and subsequent preterm birth.

### 2.4. Fetal Anomalies Pathway

#### 2.4.1. *α*-Fetoprotein and *β*-Human Chorionic Gonadotropin

Several earlier studies have identified elevated levels of *β*-human chorionic gonadotropin (hCG) and *α*-fetoprotein to be associated with an increased risk of sPTB in cervicovaginal fluids among asymptomatic pregnant women [20, 22, 31, 48]. In a prospective cohort study of 540 asymptomatic women, cervicovaginal hCG greater than 77.8 mIU/mL had a sensitivity of 87.5% (95% CI: 47.4, 97.9), specificity of 97% (86.5, 99.4), and adjusted OR = 20.0 (95% CI: 10.7, 37.5) in identifying women with sPTB at ≤34 weeks of gestation [49]. Elevated levels of *α*-fetoprotein were also associated with predicting early and late preterm births. Moawad et al. [47] conducted a nested case-control study with 127 asymptomatic women and examined *α*-fetoprotein at 24 and 28 weeks of gestation and subsequent sPTB. For *α*-fetoprotein measured at 24 weeks of gestation, elevated values (>90th percentile) were found to increase the risk of spontaneous preterm birth at <32 weeks (OR = 8.3, 95% CI: 2.2, 30.9) and for sPTB at <35 weeks (OR = 3.5, 95% CI: 1.8, 6.7). The levels of *α*-fetoprotein measured at 28 weeks of gestation were also associated with subsequent sPTB, but the effect estimates were weaker for subsequent sPTB.

### 2.5. Estrogen Metabolism Pathway

#### 2.5.1. Estriol

Estriol is the major form of circulating estrogen during pregnancy [50] and measurements of estriol from maternal saliva samples appear to correlate with maternal serum levels [51, 52]. While some earlier studies demonstrated that elevated (>2.1 ng/mL) salivary estriol was a better marker for later sPTB in asymptomatic pregnant women [53, 54], and the use of salivary estriol testing has potential advantages (i.e., noninvasive nature and ease of collection and approval by the Food and Drug Administration for use in women with singleton gestations), one major limitation of the use of saliva as a biomarker is confounding by factors such as patient activity, posture, food consumption, and diurnal variations in estriol levels [45, 55].

## 3. Use of Multiple Biochemical Markers

In addition to examining individual biochemical markers for predicting preterm birth, there is a growing interest in developing assessment tools using multiple markers. Given the heterogeneity of pathways leading to spontaneous preterm birth, multiple markers increase sensitivity of prediction by combining risk predictors that address diverse causes of spontaneous preterm birth [31]. In a nested case-control study of 2,929 women with singleton gestations, Goldenberg et al. [31] found that a positive level for at least one of three serum biomarkers (serum alkaline phosphatase, maternal serum *α*-fetoprotein, and granulocyte colony-stimulating factor) had a collective sensitivity of 81% and specificity of 78% for the prediction of spontaneous preterm birth at <32 weeks of gestation and 60% sensitivity and 73% specificity for the prediction of spontaneous preterm birth at <35 weeks of gestation.

## 4. Racial Disparities, Gene-Environment, Gene-Gene, and Epigenetics on Spontaneous Preterm Birth

Although a number of individual or set of multiple biomarkers for sPTB have been explored and examined, high intra-individual variability makes accurate prediction and subsequent prevention of preterm birth challenging. Advances in molecular biology and improved sophistication in methodology and technology of genomics have allowed for many investigators to turn their focus on the functional contributions of genetic variants to better understand preterm birth overall, but more importantly, the racial disparity of preterm birth [56–58]. According to 2010 data, preterm birth rate for non-Hispanic African American women in the United States was higher (17.1%) compared to non-Hispanic White women (10.8%) [59] and such disparity has not been explained fully by the differences in socioeconomic characteristics and maternal behaviors shown in much earlier research [3]. A growing body of literature provides emerging evidence for the role of gene-gene interactions and gene-environment interactions on preterm birth [56, 57, 60, 61] and the role of the field of epigenetics in understanding preterm birth [62]; given limitations in study design and methodology, that is, many were case-control studies where enrollment of cases and specimen collection took place at the initiation of labor, no study, thus far, has evaluated sPTB among asymptomatic women and the racial-genetic relationships on the influences of sPTB.

## 5. Conclusions

Although medical advances have improved the survival of preterm infants and the treatment of short- and long-term morbidities, little success has been attained in understanding and preventing preterm birth. Great efforts have been spent to characterize and define the utility of biologic fluids from various sources in predicting sPTB. Not only does identifying biochemical markers that are associated with sPTB allow researchers to gain a better understanding on the underlying mechanisms or pathways leading to preterm birth, but also, this valuable tool can guide in designing the most effective targeted intervention strategies aimed at women at risk for preterm birth.

Many studies have evaluated the association between individual or group biochemical marker(s) and sPTB among asymptomatic women; to date, results of a number of biochemical markers remain inconsistent. Understanding sPTB is challenging due to its multifactorial etiologies and pathophysiologic heterogeneity. Limitations from past studies on biomarkers of sPTB including the identification of appropriate study population (low risk versus high risk asymptomatic women), gestational age at the time of specimen collection, timing of collection relative to time of pregnancy outcome, and the definition of study outcome (i.e., preterm birth phenotypes and gestational age at delivery) may lead to discrepant findings. Furthermore, practicality and acceptability and maternal and fetal risks also are of great concern. In reviewing the literature, fetal fibronectin (>50 ng/mL, 24–26 weeks of gestation and beyond), thus far, is most effective in predicting sPTB in asymptomatic women. Along with its relatively high sensitivity, the required specimen collection procedure is minimally invasive and poses little to no maternal and fetal risks; the reliance of fetal fibronectin as a screening tool in clinical settings is justifiable in the management and for providing timely interventions for women with positive results. Cervical length, a biophysical marker measured by transvaginal ultrasound (not reviewed here), has been promoted by the medical community as a valuable screening tool for sPTB. While no evidence to date has suggested one marker outperforming another, fetal fibronectin and cervical length can be useful independently or jointly according to woman's a priori risk status for sPTB. Evidence in the current literature also has indicated cervical IL-6 and *α*-fetoprotein, in addition to fetal fibronectin, as potentially promising biochemical markers; however, little information on the sensitivity, specificity, and positive predictive value is known at this time to determine the clinical usefulness of these markers.

Additional research that improves understanding of the mechanisms of preterm birth is important. In addition to the development and validation of multiple biochemical markers to be used independently or in conjunction with other clinical and biophysical markers, demographic, and behavioral risk factors, future work in this area should include further refinement of study design and methodology in the evaluation of gene-gene and gene-environment interaction studies and the role of epigenetics in predicting sPTB across diverse populations.

## Figures and Tables

**Table 1:** Biochemical markers of spontaneous preterm birth among asymptomatic women by biological pathway.

Biologic pathways	Biochemical markers	Specimen type	Gestational age at specimen collection*			Definition of spontaneous preterm birth			
			Early pregnancy (<14 weeks of gestation)	Mid pregnancy (14–28 weeks of gestation)	Late pregnancy (>28 weeks of gestation)	<32 weeks	<34 weeks	<35 weeks	<37 weeks
Intrauterine infection and inflammation									
	Bacterial vaginosis			[5, 25]	[25]	[25]	[25]	[5]	[25]
	Interleukin (IL)-6	Cervical		[27, 29]				[27]	[28, 29]
		Serum		[29–31]		[31]	[30]	[31]	[28, 29]
									
	C-reactive protein	Serum		[30, 32–34]			[30, 33]		[28, 32–34]
									
	IL-1, IL-2, IL-8, and tumor necrosis factor-*α*	Cervical		[29, 34]					[29, 34]
		Serum		[29–31]			[30]	[31]	[29]
Extracellular matrix degradation									
	Fetal fibronectin	Cervicovaginal		[31, 35, 36, 38, 39, 42, 43]	[43]		[42, 44]	[31, 35, 36, 38, 39]	[43]
Fetal stress									
	Corticotropin-releasing hormone	Serum		[31, 46, 47]	[46]			[31, 47]	[46]
Fetal anomalies									
	*α*-Fetoprotein	Serum		[20, 22, 31, 47]				[20, 22, 31, 47]	
									
	*β*-Human chorionic gonadotropin	Cervicovaginal		[49]	[48]		[49]		[48]
Estrogen metabolism									
	Estriol	Salivary		[53, 54]	[53, 54]	[54]	[54]	[54]	[53, 54]

*Gestational age at specimen collection available for individual studies and not for meta-analysis or systematic reviews due to variations in timing of specimen collection for studies included in authors' pooled analysis.
